# Dataset of evidence based instruction forms for human error reduction in complex systems

**DOI:** 10.1016/j.dib.2020.105838

**Published:** 2020-06-08

**Authors:** Claudius Hammann, Christoph Krause, Anna Feldhütter

**Affiliations:** a Department of Mechanical Engineering, Institute of Nuclear Technology, Technical University of Munich, Bolzmannstreet 15, 85748 Garching, Germany; b Department of Mechanical Engineering, Institute of Ergonomics, Technical University of Munich, Bolzmannstreet 15, 85748 Garching, Germany; c Department of Aerospace and Geodesy, Institute of Flight System Dynamics, Technical University of Munich, Bolzmannstreet 15, 85748 Garching, Germany

**Keywords:** Safety, Risk quantification, Flight simulator, Standard operating procedures, Work error, Evidence based

## Abstract

The article describes the export data of a flight research simulator experiment. The data show how different forms of presentation affect the behavior of instructions for test subjects. The representation forms algorithm, image and text are examined with regard to the number of top events, error frequencies, execution times and subjectively perceived workload. For this purpose, a study was carried out with n = 93 test persons in the research flight simulator, in which the test persons had the task of landing a passenger aircraft using the autopilot with different representation forms. 14 Possible work errors with 11 different representation forms. Further, there are questionnaire answers from test persons.

Specifications table

**Table:** 

**Subject**	Safety, Risk, Reliability and Quality
**Specific subject area**	Quantification of different instruction forms with flight research simulator
**Type of data**	Table Image Graph Algorithm Figure
**How data were acquired**	Flight data: Export action of study participants form the research flight simulator with MATLAB-Software Workload: Questionnaire of the experiment
**Data format**	Raw
**Parameters for data collection**	Test persons are fire fighters and students of the technical university. The flight research simulator is technical part of Institute of Flight System Dynamics – Technical University of Munich
**Description of data collection**	There are two different data export. The first data are the result of experience exports of flight research simulator with MATLAB. The second are answers of questionnaires from the test persons after the experiment including workload tasks, sex, age, ...
**Data source location**	Germany; Garching at Munich; Technical University of Munich; Department of Aerospace and Geodesy – Institute of Flight System Dynamics – Bolzmannstreet 15, 85,748 Garching, Germany
**Data accessibility**	Hammann, Claudius; Hurst, Simon; Schmeiser, Sebastian; Wagner, Karolin, “Data of evaluation different introduction forms with research flight simulator for reduction errors”, Mendeley Data, v4. https://data.mendeley.com/datasets/xcdn5nfmjh/4
**Related research article**	C. Hammann, C. Krause, A. Feldhütter, Evaluation of algorithmic, textual and pictorial forms of representation of standard operating procedures for error reduction in complex systems. Heliyon, 6 (2020) e03291. https://doi.org/10.1016/j.heliyon.2020.e03291

## Value of the data

• The data can be used to design future instructions to make reduce errors in complex systems. The data are based on an evidence-based experiment and can therefore be transferred to other applications to answer what type of representation form are most efficient.
• The greatest benefit of the data is given by experts who create instructions for use in complex systems (nuclear reactors, control centers, aircrafts, ...). Especially, if the relationship between the probability of occurrence and the extent of the damage is very big.
• The survey of the subjects through the NASA-RTLX questionnaire allows an analysis of the guideline requirement of the day reading the instruction. The simulator data are particularly useful in the development of further methods for measuring various instructions. Direct hypotheses can be created.

## 1 Data description

The data are based on an interdisciplinary project between several institutes of the Technical University of Munich (Institute of Nuclear Technology, Institute of Ergonomics and Institute of Flight System Dynamics). The data described consists of images that describe the test setting and the flight simulator. There is also data that was exported from the simulator to quantify the flight behavior of the test subjects. The test subjects were also questioned using a questionnaire.

**Fig. 1:**
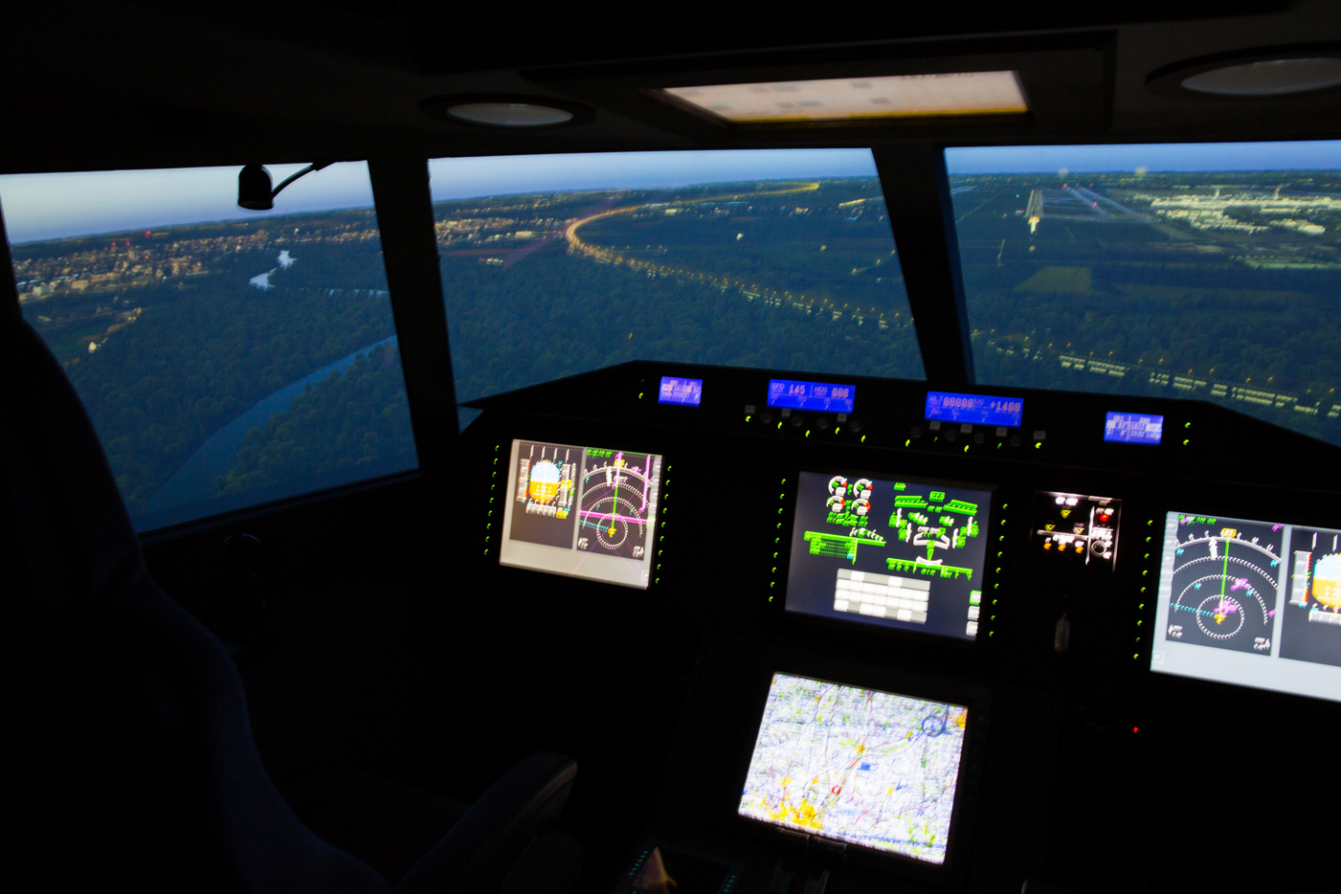

Fig. 1 shows the inside view of the flight simulator with the controls. The simulated scenario shows the approach to an airport. In the meantime, various bee units and the matching screens can be seen. The cockpit is based on the "Fairchild Dornier" aircraft.

**Fig. 2:**
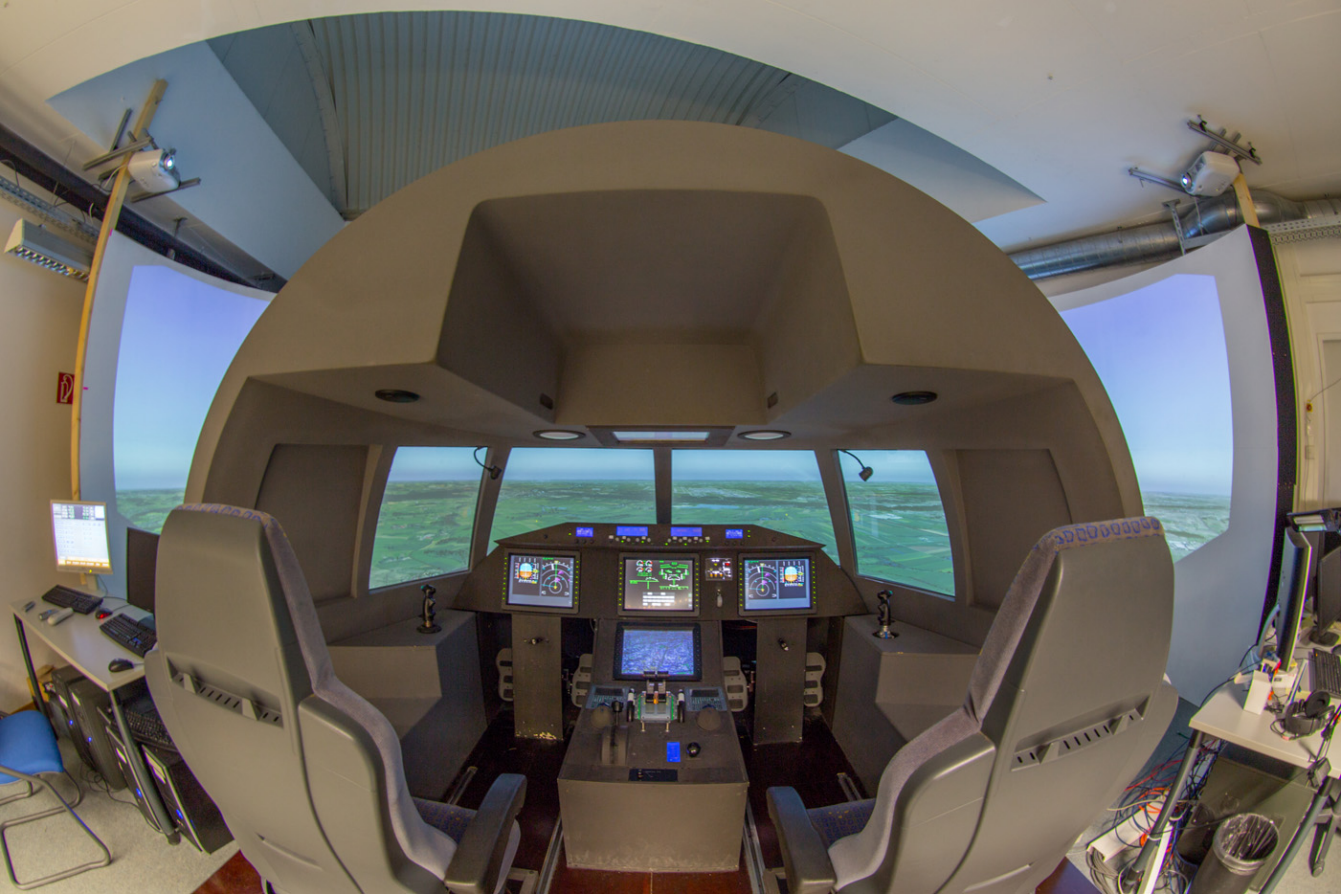

Fig. 2 shows a panorama view of the entire flight simulator. On the right and left side you can see the two control centers for the training management and in the middle the two seats for the pilots. It features a three-channel visual system with over 180° viewing angle and Full-HD resolution for each channel. The cockpit has one active side stick made by Wittenstein, which includes an electronic control loading system to allow force feedback and variable hard and soft stops.

Table 1 shows the exported simulator data of the respective test person. There is a total of 93 test persons. Each test person has been assigned a set of instructions (test, image, algorithm). The respective data show the time [s] for the respective step for landing the simulated aircraft. The metric of the data set means: t_init = time for possible start of the action, t_succ = time for conversion of the action, t_succ-t_init = required time for the action step, t_succ-t_preTask = time for conversion if time too long from previous action step. A total of 14 individual steps in the instructions were analyzed.

**Table 1:** 

Test person number	Introduction form	Metric	1. Introduction (Flaps set to 1)	2. Introduction (HDG set to 45)	3. Introduction (SPD set to 180)	4. Introduction (HDG set to 83)	5. Introduction (Gear down)	6. Introduction (Push APR button)	7. Introduction (Flaps set to 2)	8. Introduction (SPD set to 150)	9. Introduction (Flaps set to 3)	10. Introduction (SPD set to 140)	11. Introduction (Flaps set to full)	12. Introduction (Push ATHR button)	13. Introduciton (SCB set to 0)	14. Introduction (Set spoil full)
VP001	text	t_init	26.83	68.37	102.86	113.58	160.29	141.73	240.12	249.78	283.02	288.88	307.08			
VP001	text	t_succ	52.91	102.86	122.87	160.29	196.18	240.12	249.78	268.35	288.88	303.24	312.61			
VP001	text	t_succ-t_init	26.08	34.49	20.01	46.71	35.89	98.39	9.66	18.57	5.86	14.36	5.53			
VP001	text	t_succ-t_preTask	26.08	34.49	20.01	37.42	35.89	43.94	9.66	18.57	5.86	14.36	5.53			
VP002	algorithm	t_init	75.64	117.21	149.75	170.55	177.56	194.66	228.88	236.09	284.51	292.93	302.27	299.33	339.16	347.93
VP002	algorithm	t_succ	114.15	149.75	159.13	177.56	194.76	228.88	236.09	246.81	292.93	299.37	306.39	339.16	347.93	369.06
VP002	algorithm	t_succ-t_init	38.51	32.54	9.38	7.01	17.2	34.22	7.21	10.72	8.42	6.44	4.12	39.83	8.77	21.13
VP002	algorithm	t_succ-t_preTask	38.51	32.54	9.38	7.01	17.2	34.12	7.21	10.72	8.42	6.44	4.12	32.77	8.77	21.13
VP003	image	t_init	79.01	121.42	132.91	152.59	175.62	176.91	212.95	223.3	269.69	278	288.45	280.27	320.67	330.74
VP003	image	t_succ	90.06	132.91	155.53	175.62	198.18	212.95	223.3	234.35	278	293.46	301.75	320.67	330.74	342.94
VP003	image	t_succ-t_init	11.05	11.49	22.62	23.03	22.56	36.04	10.35	11.05	8.31	15.46	13.3	40.4	10.07	12.2
VP003	image	t_succ-t_preTask	11.05	11.49	22.62	20.09	22.56	14.77	10.35	11.05	8.31	15.46	13.3	18.92	10.07	12.2
VP004	algorithm	t_init	36.26	77.96	90.51	110.87	123.05	133.84	162.13	169.51	213.32	215.05	228.71	238.98	246.42	257.48
VP004	algorithm	t_succ	50.41	90.51	99.73	123.05	144.69	162.13	169.51	181.91	215.05	221.7	231.34	246.42	257.48	269.28
VP004	algorithm	t_succ-t_init	14.15	12.55	9.22	12.18	21.64	28.29	7.38	12.4	1.73	6.65	2.63	7.44	11.06	11.8
VP004	algorithm	t_succ-t_preTask	14.15	12.55	9.22	12.18	21.64	17.44	7.38	12.4	1.73	6.65	2.63	7.44	11.06	11.8
VP005	image	t_init	68.2	110.56	146.96	160.28	173.19	184.44	234.61	251.4	294.48	294.68	298.99	284.1	326.77	336.79
VP005	image	t_succ	103.21	146.96	158.83	173.19	222.93	234.61	251.4	264.3	294.68	304.59	312.94	326.77	336.79	345.98
VP005	image	t_succ-t_init	35.01	36.4	11.87	12.91	49.74	50.17	16.79	12.9	0.2	9.91	13.95	42.67	10.02	9.19
VP005	image	t_succ-t_preTask	35.01	36.4	11.87	12.91	49.74	11.68	16.79	12.9	0.2	9.91	13.95	13.83	10.02	9.19
VP006	text	t_init	24.14	66.48	72.41	94.69	99.65	115.7	121.89	129.27	170.79		186.6	226.1	225.84	234.92
VP006	text	t_succ	28.65	72.41	78.45	99.65	110.37	121.89	129.27	135.02		181.54	174.87	225.84	234.92	239.17
VP006	text	t_succ-t_init	4.51	5.93	6.04	4.96	10.72	6.19	7.38	5.75			-11.73	-0.26	9.08	4.25
VP006	text	t_succ-t_preTask	4.51	5.93	6.04	4.96	10.72	6.19	7.38	5.75			-11.73	-0.26	9.08	4.25
VP007	algorithm	t_init	17.28	59.09	90.11	107.11	125.53	133.77	197.43	208.69	252.4	257.69	269.4			
VP007	algorithm	t_succ	54.15	90.11	107.15	125.53	151.2	197.43	208.69	232.29	257.69	266.39	273.94			
VP007	algorithm	t_succ-t_init	36.87	31.02	17.04	18.42	25.67	63.66	11.26	23.6	5.29	8.7	4.54			
VP007	algorithm	t_succ-t_preTask	36.87	31.02	17.04	18.38	25.67	46.23	11.26	23.6	5.29	8.7	4.54			
VP008	image	t_init	57.4	99.76	122.94	140.71	174.31	168.63	220.64	231.29	287.38		290.32	271.89	335.52	357.68
VP008	image	t_succ	80.45	122.94	140.79	174.31	198.19	220.64	231.29	247.24		302.16	288.43	335.52	357.68	381.83
VP008	image	t_succ-t_init	23.05	23.18	17.85	33.6	23.88	52.01	10.65	15.95			-1.89	63.63	22.16	24.15
VP008	image	t_succ-t_preTask	23.05	23.18	17.85	33.52	23.88	22.45	10.65	15.95			-1.89	47.09	22.16	24.15
VP009	text	t_init	105.03	147.38	166.22	186.22	194.82	208.65	220.8	226.71	265.97	278.05	291.44	320.38	320.93	334.6
VP009	text	t_succ	116.42	166.22	178.11	194.82	210.02	220.8	226.71	237.29	278.05	284.82	293.25	320.93	334.6	354.55
VP009	text	t_succ-t_init	11.39	18.84	11.89	8.6	15.2	12.15	5.91	10.58	12.08	6.77	1.81	0.55	13.67	19.95
VP009	text	t_succ-t_preTask	11.39	18.84	11.89	8.6	15.2	10.78	5.91	10.58	12.08	6.77	1.81	0.55	13.67	19.95
VP010	image	t_init	549.39	591.7	610.35	630.08	638.3	652.7	675.88	696.87	743.11	748.11	759.64	755.68	773.88	782.68
VP010	image	t_succ	573.39	610.35	624.04	638.3	666.56	675.88	696.87	711.41	748.11	754.51	763.58	773.88	782.68	794.86
VP010	image	t_succ-t_init	24	18.65	13.69	8.22	28.26	23.18	20.99	14.54	5	6.4	3.94	18.2	8.8	12.18
VP010	image	t_succ-t_preTask	24	18.65	13.69	8.22	28.26	9.32	20.99	14.54	5	6.4	3.94	10.3	8.8	12.18
VP011	text	t_init	84.98	127.3	224.07	290.95	281.69	331.18	351.63	368.31	402.37	419.1	434.43			
VP011	text	t_succ	124.18	224.07	252.98	281.69	307.95	351.63	368.31	386.96	419.1	430.37	444.77			
VP011	text	t_succ-t_init	39.2	96.77	28.91	-9.26	26.26	20.45	16.68	18.65	16.73	11.27	10.34			
VP011	text	t_succ-t_preTask	39.2	96.77	28.91	-9.26	26.26	20.45	16.68	18.65	16.73	11.27	10.34			
VP012	algorithm	t_init	54.96	97.3	139.61	157.26	168.64	183.84	230.3	235.65	289.47	303.34	316.69		348.35	
VP012	algorithm	t_succ	82.1	139.61	154.16	168.64	213.42	230.3	235.65		303.34	310.42	319.34	348.35		
VP012	algorithm	t_succ-t_init	27.14	42.31	14.55	11.38	44.78	46.46	5.35		13.87	7.08	2.65			
VP012	algorithm	t_succ-t_preTask	27.14	42.31	14.55	11.38	44.78	16.88	5.35			7.08	2.65	29.01		
VP013	image	t_init	78.05	119.64	143.91	159.21	174.37	182.53	197.31	209.92	261.94	262.67	286.45	288.85	298.37	307.02
VP013	image	t_succ	97.99	143.91	156.99	174.37	184.62	197.31	209.92	228.46	262.67	278.99	288.18	298.37	307.02	319.86
VP013	image	t_succ-t_init	19.94	24.27	13.08	15.16	10.25	14.78	12.61	18.54	0.73	16.32	1.73	9.52	8.65	12.84
VP013	image	t_succ-t_preTask	19.94	24.27	13.08	15.16	10.25	12.69	12.61	18.54	0.73	16.32	1.73	9.52	8.65	12.84
VP014	text	t_init	102.86	145.19	165.94	181.76	199.18	206.18	265.55	272.99	324.62	318.59	332.12	302.53	351.65	367.82
VP014	text	t_succ	113.74	165.94	181.7	199.18	220.82	265.55	272.99	291.43	318.59	329.04	336.93	351.65	367.82	386.78
VP014	text	t_succ-t_init	10.88	20.75	15.76	17.42	21.64	59.37	7.44	18.44	-6.03	10.45	4.81	49.12	16.17	18.96
VP014	text	t_succ-t_preTask	10.88	20.75	15.76	17.42	21.64	44.73	7.44	18.44	-6.03	10.45	4.81	14.72	16.17	18.96
VP015	image	t_init	96.08	137.86	161.95	173.62	202.98	199.6	230.02	244.71	286.01	284.18	301.65	305.83	330.45	339.91
VP015	image	t_succ	102.75	161.95	173.76	202.98	214.4	230.02	244.71	258.6	284.18	296.29	310.9	330.45	339.91	349.02
VP015	image	t_succ-t_init	6.67	24.09	11.81	29.36	11.42	30.42	14.69	13.89	-1.83	12.11	9.25	24.62	9.46	9.11
VP015	image	t_succ-t_preTask	6.67	24.09	11.81	29.22	11.42	15.62	14.69	13.89	-1.83	12.11	9.25	19.55	9.46	9.11
VP016	algorithm	t_init	128.8	171.13	240.62	263.86	276.28	294.84	309.94	317.08	366.41	371.83	383.42			
VP016	algorithm	t_succ	142.99	240.62	253.15	276.28	293.24	309.94	317.08	339.32	371.83	380.65	397.77			
VP016	algorithm	t_succ-t_init	14.19	69.49	12.53	12.42	16.96	15.1	7.14	22.24	5.42	8.82	14.35			
VP016	algorithm	t_succ-t_preTask	14.19	69.49	12.53	12.42	16.96	15.1	7.14	22.24	5.42	8.82	14.35			
VP017	algorithm	t_init	116.71	159.01	192.97	211.28	232.63	239.26	264.92	273.55	322.25	327.94	339.2	345.87	383.49	429.03
VP017	algorithm	t_succ	132.24	192.97	209.68	232.63	246.3	264.92	273.55	291.03	327.94	334.25	344.8	383.49	429.03	438.38
VP017	algorithm	t_succ-t_init	15.53	33.96	16.71	21.35	13.67	25.66	8.63	17.48	5.69	6.31	5.6	37.62	45.54	9.35
VP017	algorithm	t_succ-t_preTask	15.53	33.96	16.71	21.35	13.67	18.62	8.63	17.48	5.69	6.31	5.6	37.62	45.54	9.35
VP018	text	t_init	41	83.42	118.88	127.19	162.94	154.94	231.35	255.14	282.68	286.94	300.24			
VP018	text	t_succ	46.51	118.88	140.3	162.94	208.67	231.35	255.14	274.75	286.94	297.9	311.09			
VP018	text	t_succ-t_init	5.51	35.46	21.42	35.75	45.73	76.41	23.79	19.61	4.26	10.96	10.85			
VP018	text	t_succ-t_preTask	5.51	35.46	21.42	22.64	45.73	22.68	23.79	19.61	4.26	10.96	10.85			
VP019	algorithm	t_init	139.66	181.3	212.04	228.05	243.36	253.26	301.01	307.87	360.21	331.43	364.91	352.88	386.94	395.97
VP019	algorithm	t_succ	157.99	212.04	227.3	243.36	288.53	301.01	307.87	320.27	331.43	346.47	370.03	386.94	395.97	406.17
VP019	algorithm	t_succ-t_init	18.33	30.74	15.26	15.31	45.17	47.75	6.86	12.4	-28.78	15.04	5.12	34.06	9.03	10.2
VP019	algorithm	t_succ-t_preTask	18.33	30.74	15.26	15.31	45.17	12.48	6.86	12.4	-28.78	15.04	5.12	16.91	9.03	10.2
VP020	image	t_init	89.88	132.19	147.46	165.68	189.47	189.64	232.06	244.65	294.55	300.14	302.49	289.34	328.5	340.24
VP020	image	t_succ	113.01	147.46	166.93	189.47	212.04	232.06	244.65	260.41	300.14	309.59	318.89	328.5	340.24	348.59
VP020	image	t_succ-t_init	23.13	15.27	19.47	23.79	22.57	42.42	12.59	15.76	5.59	9.45	16.4	39.16	11.74	8.35
VP020	image	t_succ-t_preTask	23.13	15.27	19.47	22.54	22.57	20.02	12.59	15.76	5.59	9.45	16.4	9.61	11.74	8.35
VP021	text	t_init	134.68	176.89	190.74	209.27	227.88	233.97	278.35	286.79	341.28	340.58	347.08	333.04	404.94	423.99
VP021	text	t_succ	150.11	190.74	203.47	227.88	250.04	278.35	286.79	300	340.58	349.67	358.64	404.94	423.99	463.63
VP021	text	t_succ-t_init	15.43	13.85	12.73	18.61	22.16	44.38	8.44	13.21	-0.7	9.09	11.56	71.9	19.05	39.64
VP021	text	t_succ-t_preTask	15.43	13.85	12.73	18.61	22.16	28.31	8.44	13.21	-0.7	9.09	11.56	46.3	19.05	39.64
VP022	algorithm	t_init	155.54	197.81	222.9	240.74	266.91	268.18	300.99	310.6	355.94	365.79	377.01	374.21	397.72	413.04
VP022	algorithm	t_succ	183.36	222.9	236.51	266.91	284.41	300.99	310.6	321.03	365.79	371.89	375.49	397.72	413.04	422.39
VP022	algorithm	t_succ-t_init	27.82	25.09	13.61	26.17	17.5	32.81	9.61	10.43	9.85	6.1	-1.52	23.51	15.32	9.35
VP022	algorithm	t_succ-t_preTask	27.82	25.09	13.61	26.17	17.5	16.58	9.61	10.43	9.85	6.1	-1.52	22.23	15.32	9.35
VP023	image	t_init	79.42	121.74	133.51	150.93	182.69	174.78	209.93	217.43	258.87		270.67	283.44	283.36	293.17
VP023	image	t_succ	83.64	133.51	159.27	182.69	198.64	209.93	217.43	231.54		257.6	247.45	283.36	293.17	307.32
VP023	image	t_succ-t_init	4.22	11.77	25.76	31.76	15.95	35.15	7.5	14.11			-23.22	-0.08	9.81	14.15
VP023	image	t_succ-t_preTask	4.22	11.77	25.76	23.42	15.95	11.29	7.5	14.11			-23.22	-0.08	9.81	14.15
VP024	text	t_init	42.12	84.3	99.1	118.39	128.75	141.31	157.14	163.21	203.23	207.76	220.61	251.54	253.49	259.71
VP024	text	t_succ	58.38	99.1	109.06	128.75	146.69	157.14	163.21	172.65	207.76	214.82	222.27	253.49	259.71	273.71
VP024	text	t_succ-t_init	16.26	14.8	9.96	10.36	17.94	15.83	6.07	9.44	4.53	7.06	1.66	1.95	6.22	14
VP024	text	t_succ-t_preTask	16.26	14.8	9.96	10.36	17.94	10.45	6.07	9.44	4.53	7.06	1.66	1.95	6.22	14
VP025	image	t_init	97.86	139.69	148.66	167	176.29	189.24	203.35	210.92	254.85	255.62	270.36	296.86	296.76	303.81
VP025	image	t_succ	107.79	148.66	166.45	176.29	193.09	203.35	210.92	221.26	255.62	263.69	275.36	296.76	303.81	310.67
VP025	image	t_succ-t_init	9.93	8.97	17.79	9.29	16.8	14.11	7.57	10.34	0.77	8.07	5	-0.1	7.05	6.86
VP025	image	t_succ-t_preTask	9.93	8.97	17.79	9.29	16.8	10.26	7.57	10.34	0.77	8.07	5	-0.1	7.05	6.86
VP026	text	t_init	200.14	242.14	253.1	274.44	286.61	297.18	331.09	343.62	388.54	391.52	406.93	398.39	447.61	473.01
VP026	text	t_succ	213.98	253.1	269.89	286.61	315.36	331.09	343.62	355.91	391.52	401.33	417.91	447.61	473.01	482.71
VP026	text	t_succ-t_init	13.84	10.96	16.79	12.17	28.75	33.91	12.53	12.29	2.98	9.81	10.98	49.22	25.4	9.7
VP026	text	t_succ-t_preTask	13.84	10.96	16.79	12.17	28.75	15.73	12.53	12.29	2.98	9.81	10.98	29.7	25.4	9.7
VP027	text	t_init	25.46	67.06	121.23	144.08	154.78	172.56	207.94	215.82	255.73	264.15	278.44			
VP027	text	t_succ	43.69	121.23	131.87	154.78	175.44	207.94	215.82	228.7	264.15	273.44	282.42			
VP027	text	t_succ-t_init	18.23	54.17	10.64	10.7	20.66	35.38	7.88	12.88	8.42	9.29	3.98			
VP027	text	t_succ-t_preTask	18.23	54.17	10.64	10.7	20.66	32.5	7.88	12.88	8.42	9.29	3.98			
VP028	text	t_init	24.46	66.29	106.82	120.5	137.21	146.11	166.07	174.29	215.15	198.45	228.88	257.4	258.96	276.64
VP028	text	t_succ	36.31	106.82	121.03	137.21	149.83	166.07	174.29	187.11	198.45	205.76	230.6	258.96	276.64	300.39
VP028	text	t_succ-t_init	11.85	40.53	14.21	16.71	12.62	19.96	8.22	12.82	-16.7	7.31	1.72	1.56	17.68	23.75
VP028	text	t_succ-t_preTask	11.85	40.53	14.21	16.18	12.62	16.24	8.22	12.82	-16.7	7.31	1.72	1.56	17.68	23.75
VP029	text	t_init	161.74	203.65	219.9	228.39	263.88	251.44	304.92	318.1	363.41	366.04	366.57	349.73	395.73	412.25
VP029	text	t_succ	178.62	219.9	235.98	263.88	283.81	304.92	318.1	327.97	366.04	374.25	381.84	395.73	412.25	425.24
VP029	text	t_succ-t_init	16.88	16.25	16.08	35.49	19.93	53.48	13.18	9.87	2.63	8.21	15.27	46	16.52	12.99
VP029	text	t_succ-t_preTask	16.88	16.25	16.08	27.9	19.93	21.11	13.18	9.87	2.63	8.21	15.27	13.89	16.52	12.99
VP030	algorithm	t_init	33.19	74.8	98.45	114.08	134.86	139.39	171.81	180.2	224.49	220.55	238.18	245.68	272.7	286.19
VP030	algorithm	t_succ	52.51	98.45	115.13	134.86	156.32	171.81	180.2	197.8	220.55	230.7	241.74	272.7	286.19	296.5
VP030	algorithm	t_succ-t_init	19.32	23.65	16.68	20.78	21.46	32.42	8.39	17.6	-3.94	10.15	3.56	27.02	13.49	10.31
VP030	algorithm	t_succ-t_preTask	19.32	23.65	16.68	19.73	21.46	15.49	8.39	17.6	-3.94	10.15	3.56	27.02	13.49	10.31
VP031	algorithm	t_init	90.94	133.33	192.28	213.85	233.14	245.69	278.27	286.7	332.11	333.04	346.68			
VP031	algorithm	t_succ	122.85	192.28	209.73	233.14	261.7	278.27	286.7	307.74	333.04	343.27	351.32			
VP031	algorithm	t_succ-t_init	31.91	58.95	17.45	19.29	28.56	32.58	8.43	21.04	0.93	10.23	4.64			
VP031	algorithm	t_succ-t_preTask	31.91	58.95	17.45	19.29	28.56	16.57	8.43	21.04	0.93	10.23	4.64			
VP032	image	t_init	55.45	97.6	134.99	149.5	177.02	178.85	231.11	243.53	286.78	279.49	294.24		318.94	341.57
VP032	image	t_succ	79.6	134.99	150.03	177.02	207.55	231.11	243.53	263.26	279.49	294.03	303.28	318.94	341.57	
VP032	image	t_succ-t_init	24.15	37.39	15.04	27.52	30.53	52.26	12.42	19.73	-7.29	14.54	9.04		22.63	
VP032	image	t_succ-t_preTask	24.15	37.39	15.04	26.99	30.53	23.56	12.42	19.73	-7.29	14.54	9.04	15.66	22.63	
VP033	image	t_init	42.88	84.74	99.19	116.75	133.57	140.64	168.57	181.4	225.58	244	253.43	245.11	274.81	283.05
VP033	image	t_succ	56.95	99.19	114.62	133.57	152.72	168.57	181.4	196.25	244	251.56	259.79	274.81	283.05	293.34
VP033	image	t_succ-t_init	14.07	14.45	15.43	16.82	19.15	27.93	12.83	14.85	18.42	7.56	6.36	29.7	8.24	10.29
VP033	image	t_succ-t_preTask	14.07	14.45	15.43	16.82	19.15	15.85	12.83	14.85	18.42	7.56	6.36	15.02	8.24	10.29
VP034	text	t_init	64.23	106.62		140.72	152.62	162.95	187.57	201.68	239.7	232.53	253.13	268.73	276.23	286.47
VP034	text	t_succ	76.94		133.05	152.62	175.67	187.57	201.68	212.55	232.53	241.48	249.93	276.23	286.47	296.37
VP034	text	t_succ-t_init	12.71			11.9	23.05	24.62	14.11	10.87	-7.17	8.95	-3.2	7.5	10.24	9.9
VP034	text	t_succ-t_preTask	12.71			11.9	23.05	11.9	14.11	10.87	-7.17	8.95	-3.2	7.5	10.24	9.9
VP035	algorithm	t_init	157.67	199.43	223.26	240.09	255.31	264.29	284.07	290.28	330.93		348.88	375.31	382.2	393.24
VP035	algorithm	t_succ	166.49	223.26	238.11	255.31	270.26	284.07	290.28	302.81		343.7	351.57	382.2	393.24	421.15
VP035	algorithm	t_succ-t_init	8.82	23.83	14.85	15.22	14.95	19.78	6.21	12.53			2.69	6.89	11.04	27.91
VP035	algorithm	t_succ-t_preTask	8.82	23.83	14.85	15.22	14.95	13.81	6.21	12.53			2.69	6.89	11.04	27.91
VP036	algorithm	t_init	246.06	288.38	332.07	345.63	362.15	372.27	395.8	402.29	441.94	424.39	454.2	477.6	453.12	493.49
VP036	algorithm	t_succ	259.5	332.07	343.01	362.15	378.18	395.8	402.29	414.14	424.39	432.11	440.19	453.12	493.49	508.93
VP036	algorithm	t_succ-t_init	13.44	43.69	10.94	16.52	16.03	23.53	6.49	11.85	-17.55	7.72	-14.01	-24.48	40.37	15.44
VP036	algorithm	t_succ-t_preTask	13.44	43.69	10.94	16.52	16.03	17.62	6.49	11.85	-17.55	7.72	-14.01	-24.48	40.37	15.44
VP037	text	t_init	572.19	614.44	650.18	667.35	711.92	696.4	742.84	751.3	791.48	789.24	803.89			
VP037	text	t_succ	584.37	650.18	694.54	711.92	728.64	742.84	751.3	761.41	789.24	801.1	814.69			
VP037	text	t_succ-t_init	12.18	35.74	44.36	44.57	16.72	46.44	8.46	10.11	-2.24	11.86	10.8			
VP037	text	t_succ-t_preTask	12.18	35.74	44.36	17.38	16.72	14.2	8.46	10.11	-2.24	11.86	10.8			
VP038	image	t_init	646.65	688.49	700.96	716.92	736.87	740.47	765.81	777.64	816.72	824.48	841.59	845.52	853.52	854.82
VP038	image	t_succ	654.28	700.96	719.94	736.87	748.84	765.81	777.64		824.48	835.94	846.39	853.52	854.82	883.08
VP038	image	t_succ-t_init	7.63	12.47	18.98	19.95	11.97	25.34	11.83		7.76	11.46	4.8	8	1.3	28.26
VP038	image	t_succ-t_preTask	7.63	12.47	18.98	16.93	11.97	16.97	11.83			11.46	4.8	7.13	1.3	28.26
VP039	image	t_init	440.3	487.01	495.93	517.25	538.54	542.58	584.17	596.71	633.07	630.06	646.95	646.75	659.93	670.4
VP039	image	t_succ	450.07	495.93	514.32	538.54	559.53	584.17	596.71	616.53	630.06	641.12	648.85	659.93	670.4	679.27
VP039	image	t_succ-t_init	9.77	8.92	18.39	21.29	20.99	41.59	12.54	19.82	-3.01	11.06	1.9	13.18	10.47	8.87
VP039	image	t_succ-t_preTask	9.77	8.92	18.39	21.29	20.99	24.64	12.54	19.82	-3.01	11.06	1.9	11.08	10.47	8.87
VP040	text	t_init	103.76	145.2	171.3	191.28	201.08	214.43	262.47	275.74	322.18	312.68	327.4	312.92	349.41	361.48
VP040	text	t_succ	120	171.3	180.96	201.08	228.81	262.47	275.74	288.08	312.68	321.57	332.39	349.41	361.48	372.28
VP040	text	t_succ-t_init	16.24	26.1	9.66	9.8	27.73	48.04	13.27	12.34	-9.5	8.89	4.99	36.49	12.07	10.8
VP040	text	t_succ-t_preTask	16.24	26.1	9.66	9.8	27.73	33.66	13.27	12.34	-9.5	8.89	4.99	17.02	12.07	10.8
VP041	text	t_init	50.85	92.93	104.43	123.14	131.36	144.86	198.81	206.06	258.09	241.26	265.85	239.23	280.31	291.76
VP041	text	t_succ	64.07	104.43	116.48	131.36	170.3	198.81	206.06	214.45	241.26	246.31	258.8	280.31	291.76	321.31
VP041	text	t_succ-t_init	13.22	11.5	12.05	8.22	38.94	53.95	7.25	8.39	-16.83	5.05	-7.05	41.08	11.45	29.55
VP041	text	t_succ-t_preTask	13.22	11.5	12.05	8.22	38.94	28.51	7.25	8.39	-16.83	5.05	-7.05	21.51	11.45	29.55
VP042	algorithm	t_init	52.22	93.78	161.19	189.44	200.86	220.24	281.2	290.64	321.56	326.36	354.52			
VP042	algorithm	t_succ	61.63	161.19	176.36	200.86	252.83	281.2	290.64	306.41	326.36	349.41	356.95			
VP042	algorithm	t_succ-t_init	9.41	67.41	15.17	11.42	51.97	60.96	9.44	15.77	4.8	23.05	2.43			
VP042	algorithm	t_succ-t_preTask	9.41	67.41	15.17	11.42	51.97	28.37	9.44	15.77	4.8	23.05	2.43			
VP043	text	t_init	32.99	74.45	94.15	113.79	124.84	136.77	154.82	160.75	198.21	200.24	213.6	248.43	249.27	268.5
VP043	text	t_succ	50.01	94.15	105.63	124.84	141.23	154.82	160.75	170.45	200.24	207.45	218.65	249.27	268.5	280.04
VP043	text	t_succ-t_init	17.02	19.7	11.48	11.05	16.39	18.05	5.93	9.7	2.03	7.21	5.05	0.84	19.23	11.54
VP043	text	t_succ-t_preTask	17.02	19.7	11.48	11.05	16.39	13.59	5.93	9.7	2.03	7.21	5.05	0.84	19.23	11.54
VP044	image	t_init	43.52	85.86	105.41	124.82	132.34	147.31	150.93	165.64	206.13	194.59	220.28	259.36	259.1	269.48
VP044	image	t_succ	59.1	105.41	118.22	132.34	141.86	150.93	165.64	176.92	194.59	202.82	220.08	259.1	269.48	277.23
VP044	image	t_succ-t_init	15.58	19.55	12.81	7.52	9.52	3.62	14.71	11.28	-11.54	8.23	-0.2	-0.26	10.38	7.75
VP044	image	t_succ-t_preTask	15.58	19.55	12.81	7.52	9.52	3.62	14.71	11.28	-11.54	8.23	-0.2	-0.26	10.38	7.75
VP045	algorithm	t_init	115.04	157.39	174.83	191.19	218.91	216.38	277.11	288.91	335.78	324.73	343.4	313.06	372.66	395.63
VP045	algorithm	t_succ	124.3	174.83	192.79	218.91	234.51	277.11	288.91		324.73	338.07	351.44	372.66	395.63	409.92
VP045	algorithm	t_succ-t_init	9.26	17.44	17.96	27.72	15.6	60.73	11.8		-11.05	13.34	8.04	59.6	22.97	14.29
VP045	algorithm	t_succ-t_preTask	9.26	17.44	17.96	26.12	15.6	42.6	11.8			13.34	8.04	21.22	22.97	14.29
VP046	text	t_init	142.39	184.79	205.7	223.53	232.52	246.07	252.07	256.13		355.41		346.11		
VP046	text	t_succ	151.69	205.7	219.4	232.52		252.07	256.13	310.38	355.41					
VP046	text	t_succ-t_init	9.3	20.91	13.7	8.99		6	4.06	54.25						
VP046	text	t_succ-t_preTask	9.3	20.91	13.7	8.99		6	4.06	54.25	45.03					
VP047	algorithm	t_init	26.8	69.13	77.21	98.9	104.73	120.42	146.61	156.73	203.82	207.33	220.16	222.06	260.79	274.62
VP047	algorithm	t_succ	31.63	77.21	85.08	104.73	119.47	146.61	156.73	171.47	207.33	215.52	224	260.79	274.62	286.62
VP047	algorithm	t_succ-t_init	4.83	8.08	7.87	5.83	14.74	26.19	10.12	14.74	3.51	8.19	3.84	38.73	13.83	12
VP047	algorithm	t_succ-t_preTask	4.83	8.08	7.87	5.83	14.74	26.19	10.12	14.74	3.51	8.19	3.84	36.79	13.83	12
VP048	text	t_init	88.88	131.3	141.18	159.73	172.66	181.91	190.09	194.7	291.2	304.24	297.39	283.4	329.53	339.64
VP048	text	t_succ	106.36	141.18	158.4	172.66		190.09	194.7	208.06	304.24	309.8	316.54	329.53	339.64	363.09
VP048	text	t_succ-t_init	17.48	9.88	17.22	12.93		8.18	4.61	13.36	13.04	5.56	19.15	46.13	10.11	23.45
VP048	text	t_succ-t_preTask	17.48	9.88	17.22	12.93		8.18	4.61	13.36	13.04	5.56	19.15	12.99	10.11	23.45
VP049	text	t_init	20.86	55.18	50.15	75.58	88.04	96.81	107.22	112.77	188.06	175.7	201.64	201.94	211.02	220.75
VP049	text	t_succ	29.52	50.15	66.89	88.04		107.22	112.77	122.9	175.7	181.57	200.41	211.02	220.75	243.78
VP049	text	t_succ-t_init	8.66	-5.03	16.74	12.46		10.41	5.55	10.13	-12.36	5.87	-1.23	9.08	9.73	23.03
VP049	text	t_succ-t_preTask	8.66	-5.03	16.74	12.46		10.41	5.55	10.13	-12.36	5.87	-1.23	9.08	9.73	23.03
VP050	image	t_init	108.97	148.99	148.1	171.35	178.56	192.51	235.32	243.67	298.83	297.87	304.93	289.78	328.81	346.11
VP050	image	t_succ	122.33	148.1	163.69	178.56	198.8	235.32	243.67	254.84	297.87	306.71	314.65	328.81	346.11	354.41
VP050	image	t_succ-t_init	13.36	-0.89	15.59	7.21	20.24	42.81	8.35	11.17	-0.96	8.84	9.72	39.03	17.3	8.3
VP050	image	t_succ-t_preTask	13.36	-0.89	15.59	7.21	20.24	36.52	8.35	11.17	-0.96	8.84	9.72	14.16	17.3	8.3
VP051	text	t_init	100.89	143.22	164.87	180.73	224.58	205.42	264.24	270.82	321.81	321.46	329.42			
VP051	text	t_succ	113.43	164.87	210.18	224.58	248.37	264.24	270.82	281	321.46	328.45	340.31			
VP051	text	t_succ-t_init	12.54	21.65	45.31	43.85	23.79	58.82	6.58	10.18	-0.35	6.99	10.89			
VP051	text	t_succ-t_preTask	12.54	21.65	45.31	14.4	23.79	15.87	6.58	10.18	-0.35	6.99	10.89			
VP052	algorithm	t_init	121.49	162.67	221.9	240.57	260.06	274.19	304.21	312.5	350.52	358.15	369.02			
VP052	algorithm	t_succ	164.86	221.9	237.83	260.06	279.6	304.21	312.5	328.99	358.15	364.58	376.19			
VP052	algorithm	t_succ-t_init	43.37	59.23	15.93	19.49	19.54	30.02	8.29	16.49	7.63	6.43	7.17			
VP052	algorithm	t_succ-t_preTask	43.37	57.04	15.93	19.49	19.54	24.61	8.29	16.49	7.63	6.43	7.17			
VP053	text	t_init	17.67	58.7	121.82	142.88	150.58	171.27	182.71	189.64	226.22	215.15	237.63	299.11	255.59	270.89
VP053	text	t_succ	37.96	121.82	134.47	150.58	167.95	182.71	189.64	202.9	215.15	226.29	236.63	255.59	270.89	282.99
VP053	text	t_succ-t_init	20.29	63.12	12.65	7.7	17.37	11.44	6.93	13.26	-11.07	11.14	-1	-43.52	15.3	12.1
VP053	text	t_succ-t_preTask	20.29	63.12	12.65	7.7	17.37	11.44	6.93	13.26	-11.07	11.14	-1	-43.52	15.3	12.1
VP054	image	t_init	95.53	134.66	142.98	160.76	168.33	182.42	197.92	203.2	243.62	248.34	260.66	290.68	290.45	300.14
VP054	image	t_succ	104.37	142.98	155.06	168.33	186.52	197.92	203.2	214.32	248.34	255.35	265.5	290.45	300.14	312.14
VP054	image	t_succ-t_init	8.84	8.32	12.08	7.57	18.19	15.5	5.28	11.12	4.72	7.01	4.84	-0.23	9.69	12
VP054	image	t_succ-t_preTask	8.84	8.32	12.08	7.57	18.19	11.4	5.28	11.12	4.72	7.01	4.84	-0.23	9.69	12
VP055	algorithm	t_init	61.14	102.22	114.9	136.09	145.47	159.01	163.23	167.89	211.08	211.75	224.99	271.32	271.95	312.84
VP055	algorithm	t_succ	84.12	114.9	127.37	145.47	152.7	163.23	167.89	185.77	211.75	223.11	228.25	271.95	312.84	327.52
VP055	algorithm	t_succ-t_init	22.98	12.68	12.47	9.38	7.23	4.22	4.66	17.88	0.67	11.36	3.26	0.63	40.89	14.68
VP055	algorithm	t_succ-t_preTask	22.98	12.68	12.47	9.38	7.23	4.22	4.66	17.88	0.67	11.36	3.26	0.63	40.89	14.68
VP056	text	t_init	31.04	72.14	79.61	96.4	126.06	120.33	161.84	169.4	223.91	229	232.13	219.39	272.84	284.54
VP056	text	t_succ	45.17	79.61	98.68	126.06	143.82	161.84	169.4	183.2	229	236.84	243.29	272.84	284.54	294.5
VP056	text	t_succ-t_init	14.13	7.47	19.07	29.66	17.76	41.51	7.56	13.8	5.09	7.84	11.16	53.45	11.7	9.96
VP056	text	t_succ-t_preTask	14.13	7.47	19.07	27.38	17.76	18.02	7.56	13.8	5.09	7.84	11.16	29.55	11.7	9.96
VP057	image	t_init	34.27	76.46	84.13	104.13	109.98	125.32	137.78	146.03	190.47	199.54	214.56	232.47	232.37	242.17
VP057	image	t_succ	44.25	84.13	95.4	109.98	123.53	137.78	146.03	156.73	199.54	208.27	218.58	232.37	242.17	252.63
VP057	image	t_succ-t_init	9.98	7.67	11.27	5.85	13.55	12.46	8.25	10.7	9.07	8.73	4.02	-0.1	9.8	10.46
VP057	image	t_succ-t_preTask	9.98	7.67	11.27	5.85	13.55	12.46	8.25	10.7	9.07	8.73	4.02	-0.1	9.8	10.46
VP058	algorithm	t_init	11.99	54.07	65.82	84.82	92.23	106.78	132.36	140.59	189.53	202.04	206.26	209.32	228.95	237.08
VP058	algorithm	t_succ	16.9	65.82	77.47	92.23	109.24	132.36	140.59	152.66	202.04	196.76	210.44	228.95	237.08	257.63
VP058	algorithm	t_succ-t_init	4.91	11.75	11.65	7.41	17.01	25.58	8.23	12.07	12.51	-5.28	4.18	19.63	8.13	20.55
VP058	algorithm	t_succ-t_preTask	4.91	11.75	11.65	7.41	17.01	23.12	8.23	12.07	12.51	-5.28	4.18	18.51	8.13	20.55
VP059	image	t_init	29.65	64.71	72.75	88.58	98.37	109.52	119.56	127.24	169.39	178.54	192.43	220.01	219.81	227.58
VP059	image	t_succ	38.95	72.75	84.3	98.37	109.41	119.56	127.24	138.57	178.54	186.42	195.24	219.81	227.58	236.13
VP059	image	t_succ-t_init	9.3	8.04	11.55	9.79	11.04	10.04	7.68	11.33	9.15	7.88	2.81	-0.2	7.77	8.55
VP059	image	t_succ-t_preTask	9.3	8.04	11.55	9.79	11.04	10.04	7.68	11.33	9.15	7.88	2.81	-0.2	7.77	8.55
VP060	image	t_init	54.83	97.06	105.86	124.74	147.73	148.89	183.91	194.64	241	248.83	259.67	251.38	272.36	282.63
VP060	image	t_succ	66.35	105.86	123.1	147.73	174.52	183.91	194.64	206.65	248.83	256.82	263.64	272.36	282.63	290.05
VP060	image	t_succ-t_init	11.52	8.8	17.24	22.99	26.79	35.02	10.73	12.01	7.83	7.99	3.97	20.98	10.27	7.42
VP060	image	t_succ-t_preTask	11.52	8.8	17.24	22.99	26.79	9.39	10.73	12.01	7.83	7.99	3.97	8.72	10.27	7.42
VP061	algorithm	t_init	21.95	63.82	84.26	103.93	117.45	128.23	153.14	158.26	201.65	206.61	219.85	236.72	255.41	263.1
VP061	algorithm	t_succ	34.13	84.26	96.07	117.45	139.29	153.14	158.26	174.43	206.61	215.45	225.22	255.41	263.1	277.56
VP061	algorithm	t_succ-t_init	12.18	20.44	11.81	13.52	21.84	24.91	5.12	16.17	4.96	8.84	5.37	18.69	7.69	14.46
VP061	algorithm	t_succ-t_preTask	12.18	20.44	11.81	13.52	21.84	13.85	5.12	16.17	4.96	8.84	5.37	18.69	7.69	14.46
VP062	image	t_init	46.65	88.91	106.17	127.13	136.57	149.37	158.61	168.34	207.93	211.33	224.93	261.08	260.63	268.28
VP062	image	t_succ	79.94	106.17	115.41	136.57	147.03	158.61	168.34	177.54	211.33	218.65	226.53	260.63	268.28	274.62
VP062	image	t_succ-t_init	33.29	17.26	9.24	9.44	10.46	9.24	9.73	9.2	3.4	7.32	1.6	-0.45	7.65	6.34
VP062	image	t_succ-t_preTask	33.29	17.26	9.24	9.44	10.46	9.24	9.73	9.2	3.4	7.32	1.6	-0.45	7.65	6.34
VP063	algorithm	t_init	52.07	94.37	108.31	131.32	141.83	154.24	169.95	182.08	230.9	240.78	260.05	259.9	304.89	326.76
VP063	algorithm	t_succ	67.74	108.31	118.84	141.83	156.92	169.95	182.08	200.7	240.78	251.38	265.43	304.89	326.76	332.97
VP063	algorithm	t_succ-t_init	15.67	13.94	10.53	10.51	15.09	15.71	12.13	18.62	9.88	10.6	5.38	44.99	21.87	6.21
VP063	algorithm	t_succ-t_preTask	15.67	13.94	10.53	10.51	15.09	13.03	12.13	18.62	9.88	10.6	5.38	39.46	21.87	6.21
VP064	image	t_init	84.51	126.82	135.17	154.14	163.88	175.88	204.13	215.77	261.09	260.23	275.9	276.96	296.85	308.07
VP064	image	t_succ	96.17	135.17	151.54	163.88	185	204.13	215.77	231.01	260.23	269.94	283.26	296.85	308.07	317.93
VP064	image	t_succ-t_init	11.66	8.35	16.37	9.74	21.12	28.25	11.64	15.24	-0.86	9.71	7.36	19.89	11.22	9.86
VP064	image	t_succ-t_preTask	11.66	8.35	16.37	9.74	21.12	19.13	11.64	15.24	-0.86	9.71	7.36	13.59	11.22	9.86
VP065	algorithm	t_init	91.09	133.51	144.77	166.67	180.67	189.59	202.63	217.15	265.87	262.6	303.89	294.54	298.38	309.7
VP065	algorithm	t_succ	109.86	144.77	162.2	180.67	187.25	202.63	217.15	234.51	262.6		280.35	298.38	309.7	315.69
VP065	algorithm	t_succ-t_init	18.77	11.26	17.43	14	6.58	13.04	14.52	17.36	-3.27		-23.54	3.84	11.32	5.99
VP065	algorithm	t_succ-t_preTask	18.77	11.26	17.43	14	6.58	13.04	14.52	17.36	-3.27		-23.54	3.84	11.32	5.99
VP066	image	t_init	110.94	151.32	153.18	173.85	202.13	197.62	248.75	260.48	307.93	311.63	310.96	293.47	345.59	358.87
VP066	image	t_succ	122.19	153.18	166.27	202.13	226.75	248.75	260.48	276.96	311.63	323.24	331.78	345.59	358.87	367.45
VP066	image	t_succ-t_init	11.25	1.86	13.09	28.28	24.62	51.13	11.73	16.48	3.7	11.61	20.82	52.12	13.28	8.58
VP066	image	t_succ-t_preTask	11.25	1.86	13.09	28.28	24.62	22	11.73	16.48	3.7	11.61	20.82	13.81	13.28	8.58
VP067	algorithm	t_init	103.62	145.29	167.09	186.94	213.98	214.47	252.17	264.06	310.83		327.3	318.21	378.15	392.81
VP067	algorithm	t_succ	112.62	167.09	183.29	213.98	235.98	252.17	264.06	276.26		334.03	318.23	378.15	392.81	400.06
VP067	algorithm	t_succ-t_init	9	21.8	16.2	27.04	22	37.7	11.89	12.2			-9.07	59.94	14.66	7.25
VP067	algorithm	t_succ-t_preTask	9	21.8	16.2	27.04	22	16.19	11.89	12.2			-9.07	59.92	14.66	7.25
VP068	image	t_init	128.41	170.13	178.95	199.82	217.01	223.28	246.41	255.37	295.41	304.08	314.48	330.41	333.7	340.67
VP068	image	t_succ	139.74	178.95	198.02	217.01	231.54	246.41	255.37	266.17	304.08	310.39	323.23	333.7	340.67	352.42
VP068	image	t_succ-t_init	11.33	8.82	19.07	17.19	14.53	23.13	8.96	10.8	8.67	6.31	8.75	3.29	6.97	11.75
VP068	image	t_succ-t_preTask	11.33	8.82	19.07	17.19	14.53	14.87	8.96	10.8	8.67	6.31	8.75	3.29	6.97	11.75
VP069	algorithm	t_init	340.12	382.4	424.83	444.48	460.66	471.6	497.48	502.39	546.32	548.89	564.44	584.77	590.4	630.23
VP069	algorithm	t_succ	373.85	424.83	439.08	460.66	484.82	497.48	502.39	516.48	548.89		568.54	590.4	630.23	643.53
VP069	algorithm	t_succ-t_init	33.73	42.43	14.25	16.18	24.16	25.88	4.91	14.09	2.57		4.1	5.63	39.83	13.3
VP069	algorithm	t_succ-t_preTask	33.73	42.43	14.25	16.18	24.16	12.66	4.91	14.09	2.57		4.1	5.63	39.83	13.3
VP070	text	t_init	430.44	471.93	529.23	549.63	576.85	586.03	628.83	667.18	702.07	710.48	728.64			
VP070	text	t_succ	451.01	529.23	548.28	576.85	599.74	628.83	667.18	690.97	710.48	724.9	757.57			
VP070	text	t_succ-t_init	20.57	57.3	19.05	27.22	22.89	42.8	38.35	23.79	8.41	14.42	28.93			
VP070	text	t_succ-t_preTask	20.57	57.3	19.05	27.22	22.89	29.09	38.35	23.79	8.41	14.42	28.93			
VP071	algorithm	t_init	147.28	188.84	221.45	234.61	278.93	263.28	331.57	344.86	382	392.04	406.34			
VP071	algorithm	t_succ	167.82	221.45	240.63	278.93	308.41	331.57	344.86	370.22	392.04	403.53	420.3			
VP071	algorithm	t_succ-t_init	20.54	32.61	19.18	44.32	29.48	68.29	13.29	25.36	10.04	11.49	13.96			
VP071	algorithm	t_succ-t_preTask	20.54	32.61	19.18	38.3	29.48	23.16	13.29	25.36	10.04	11.49	13.96			
VP072	image	t_init	8.72	51.16	59.28	80.72	88.35	102.11	108.76	117.86	161.8	171.78	191.53	210.45	211.7	228.56
VP072	image	t_succ	12.01	59.28	69.29	88.35	100.3	108.76	117.86	128.77	171.78	184.36	165.98	211.7	228.56	241.13
VP072	image	t_succ-t_init	3.29	8.12	10.01	7.63	11.95	6.65	9.1	10.91	9.98	12.58	-25.55	1.25	16.86	12.57
VP072	image	t_succ-t_preTask	3.29	8.12	10.01	7.63	11.95	6.65	9.1	10.91	9.98	12.58	-25.55	1.25	16.86	12.57
VP073	image	t_init	91.3	133.72	140.98	160.91	176.81	183.81	205.35	217.84	264.7		286.05	287.03	303.51	311.09
VP073	image	t_succ	96.54	140.98	160.75	176.81	189.92	205.35	217.84	233.29		280.77	270.82	303.51	311.09	320.52
VP073	image	t_succ-t_init	5.24	7.26	19.77	15.9	13.11	21.54	12.49	15.45			-15.23	16.48	7.58	9.43
VP073	image	t_succ-t_preTask	5.24	7.26	19.77	15.9	13.11	15.43	12.49	15.45			-15.23	16.48	7.58	9.43
VP074	algorithm	t_init	137.81	180.16	235.23	255.65	279.15	289.35	361.45	373.05	405.3	414.35	432.08			
VP074	algorithm	t_succ	173.32	235.23	251.26	279.15	337.73	361.45	373.05	392.57	414.35	426.98	442.89			
VP074	algorithm	t_succ-t_init	35.51	55.07	16.03	23.5	58.58	72.1	11.6	19.52	9.05	12.63	10.81			
VP074	algorithm	t_succ-t_preTask	35.51	55.07	16.03	23.5	58.58	23.72	11.6	19.52	9.05	12.63	10.81			
VP075	text	t_init	89.9	128.96	128.36	150.7	168.3	173.16	251.79	257.86	304.38	286.86	310.75	279.99	307.49	320.95
VP075	text	t_succ	102.22	128.36	141.62	168.3	192.36	251.79	257.86	272.61	286.86	293.66	300	307.49	320.95	330.71
VP075	text	t_succ-t_init	12.32	-0.6	13.26	17.6	24.06	78.63	6.07	14.75	-17.52	6.8	-10.75	27.5	13.46	9.76
VP075	text	t_succ-t_preTask	12.32	-0.6	13.26	17.6	24.06	59.43	6.07	14.75	-17.52	6.8	-10.75	7.49	13.46	9.76
VP076	text	t_init	73.67	115.81	124.85	142.67	157.71	165.3	215.02	224.91	274.41	261.01	281.28	260.03	303.15	321.9
VP076	text	t_succ	93.97	124.85	139.47	157.71	178.97	215.02	224.91	236.87	261.01	267.63	281.65	303.15	321.9	328.85
VP076	text	t_succ-t_init	20.3	9.04	14.62	15.04	21.26	49.72	9.89	11.96	-13.4	6.62	0.37	43.12	18.75	6.95
VP076	text	t_succ-t_preTask	20.3	9.04	14.62	15.04	21.26	36.05	9.89	11.96	-13.4	6.62	0.37	21.5	18.75	6.95
VP077	algorithm	t_init	108.3	150.01	212.3	230.48	280.96	279.7	391.78	413.43	460.26		501.07			
VP077	algorithm	t_succ	129.78	212.3	235.9	280.96	304.42	391.78	413.43	453.06		498.74	486.72			
VP077	algorithm	t_succ-t_init	21.48	62.29	23.6	50.48	23.46	112.08	21.65	39.63			-14.35			
VP077	algorithm	t_succ-t_preTask	21.48	62.29	23.6	45.06	23.46	87.36	21.65	39.63			-14.35			
VP078	image	t_init	63.91	97.44	90.55	117.58	123.93	138.36	160.99	171.75	213.54	214.24	228.42	246.31	238.51	249.17
VP078	image	t_succ	71.05	90.55	109.65	123.93	142.82	160.99	171.75	184.69	214.24	223.67	229.72	238.51	249.17	255.6
VP078	image	t_succ-t_init	7.14	-6.89	19.1	6.35	18.89	22.63	10.76	12.94	0.7	9.43	1.3	-7.8	10.66	6.43
VP078	image	t_succ-t_preTask	7.14	-6.89	19.1	6.35	18.89	18.17	10.76	12.94	0.7	9.43	1.3	-7.8	10.66	6.43
VP079	image	t_init	45.74	87.25	145.35	163.35	188.27	197.29	224.24	254.19	290.29	282.41	299			
VP079	image	t_succ	81.85	145.35	163.45	188.27	202.71	224.24	254.19	271.29	282.41	295.44	316.73			
VP079	image	t_succ-t_init	36.11	58.1	18.1	24.92	14.44	26.95	29.95	17.1	-7.88	13.03	17.73			
VP079	image	t_succ-t_preTask	36.11	58.1	18.1	24.82	14.44	21.53	29.95	17.1	-7.88	13.03	17.73			
VP080	image	t_init	67.74	109.39	113.14	135.33	143.07	156.35	194.11	201.24	261.18	218.19	263.8	254.04	239.45	247.64
VP080	image	t_succ	77.05	113.14	126.67	143.07	183.22	194.11	201.24	211.58	218.19	226.64	232.74	239.45	247.64	258.27
VP080	image	t_succ-t_init	9.31	3.75	13.53	7.74	40.15	37.76	7.13	10.34	-42.99	8.45	-31.06	-14.59	8.19	10.63
VP080	image	t_succ-t_preTask	9.31	3.75	13.53	7.74	40.15	10.89	7.13	10.34	-42.99	8.45	-31.06	-14.59	8.19	10.63
VP081	algorithm	t_init	111.4	153.78	182.3	198.08	250.4	226.04	298.87	308.61	346.74		370.44			
VP081	algorithm	t_succ	123.93	182.3	202.43	250.4	264.86	298.87	308.61	326.17		367.64	347.96			
VP081	algorithm	t_succ-t_init	12.53	28.52	20.13	52.32	14.46	72.83	9.74	17.56			-22.48			
VP081	algorithm	t_succ-t_preTask	12.53	28.52	20.13	47.97	14.46	34.01	9.74	17.56			-22.48			
VP082	text	t_init	53.93	95.68	108.59	127.37	136.17	149.46	170.35	176.41	217.61	219.72	233.72	257.29	259.79	283.02
VP082	text	t_succ	65.63	108.59	122.06	136.17	151.5	170.35	176.41	186.53	219.72	228.34	239.11	259.79	283.02	289.87
VP082	text	t_succ-t_init	11.7	12.91	13.47	8.8	15.33	20.89	6.06	10.12	2.11	8.62	5.39	2.5	23.23	6.85
VP082	text	t_succ-t_preTask	11.7	12.91	13.47	8.8	15.33	18.85	6.06	10.12	2.11	8.62	5.39	2.5	23.23	6.85
VP083	text	t_init	120.31	163.9	194.35	209.09	266.26	238.12	379.51	395.46	426.39	434.92	450.28			
VP083	text	t_succ	137.98	194.35	212.81	266.26	305.72	379.51	395.46	415.7	434.92	445.9	475.64			
VP083	text	t_succ-t_init	17.67	30.45	18.46	57.17	39.46	141.39	15.95	20.24	8.53	10.98	25.36			
VP083	text	t_succ-t_preTask	17.67	30.45	18.46	53.45	39.46	73.79	15.95	20.24	8.53	10.98	25.36			
VP084	algorithm	t_init	91.17	134.43	188.77	213.26	224.22	240.64	250.9	261.56	303.35	310.6	323.67			
VP084	algorithm	t_succ	132.41	188.77	200.14	224.22	234.1	250.9	261.56	275.88	310.6	320.82	331.5			
VP084	algorithm	t_succ-t_init	41.24	54.34	11.37	10.96	9.88	10.26	10.66	14.32	7.25	10.22	7.83			
VP084	algorithm	t_succ-t_preTask	41.24	54.34	11.37	10.96	9.88	10.26	10.66	14.32	7.25	10.22	7.83			
VP085	algorithm	t_init	105.62	148.97	182.25	199.18	220.91	226.52	256.43	262.95	312.38	313.08	325.58	332.39	361.72	375.51
VP085	algorithm	t_succ	126.99	182.25	198.01	220.91	240.5	256.43	262.95	283.4	313.08	320.3	330.07	361.72	375.51	385.87
VP085	algorithm	t_succ-t_init	21.37	33.28	15.76	21.73	19.59	29.91	6.52	20.45	0.7	7.22	4.49	29.33	13.79	10.36
VP085	algorithm	t_succ-t_preTask	21.37	33.28	15.76	21.73	19.59	15.93	6.52	20.45	0.7	7.22	4.49	29.33	13.79	10.36
VP086	text	t_init	173.99	217.7	237.85	257.15	271.82	280.96	317.08	324.3	365.35	375.04	391.31	385.6	414.67	427.13
VP086	text	t_succ	184.7	237.85	252.48	271.82	295.15	317.08	324.3	338.85	375.04	383.82	370.94	414.67	427.13	448.58
VP086	text	t_succ-t_init	10.71	20.15	14.63	14.67	23.33	36.12	7.22	14.55	9.69	8.78	-20.37	29.07	12.46	21.45
VP086	text	t_succ-t_preTask	10.71	20.15	14.63	14.67	23.33	21.93	7.22	14.55	9.69	8.78	-20.37	29.07	12.46	21.45
VP087	image	t_init	78.89	122.55	153.45	170.62	191.28	197.75	242.48	254.2	296.72	304.25	309.8	302.33	360.97	377.94
VP087	image	t_succ	97.27	153.45	170.96	191.28	226.59	242.48	254.2	272.61	304.25	318.82	332.79	360.97	377.94	393.18
VP087	image	t_succ-t_init	18.38	30.9	17.51	20.66	35.31	44.73	11.72	18.41	7.53	14.57	22.99	58.64	16.97	15.24
VP087	image	t_succ-t_preTask	18.38	30.9	17.51	20.32	35.31	15.89	11.72	18.41	7.53	14.57	22.99	28.18	16.97	15.24
VP088	image	t_init	86.4	121.97	119.02	142.35	159.13	163.75	190.71	200.97	236.07	240.32	253.97	271.01	267.87	281.97
VP088	image	t_succ	96.18	119.02	136.3	159.13	169.88	190.71	200.97	218.14	240.32	250.6	258.21	267.87	281.97	293.77
VP088	image	t_succ-t_init	9.78	-2.95	17.28	16.78	10.75	26.96	10.26	17.17	4.25	10.28	4.24	-3.14	14.1	11.8
VP088	image	t_succ-t_preTask	9.78	-2.95	17.28	16.78	10.75	20.83	10.26	17.17	4.25	10.28	4.24	-3.14	14.1	11.8
VP089	image	t_init	85.09	128.71	140.77	159.99	175.21	183.46	223.72	235.43	275.92	269.73	288.01	284.17	304.27	312.71
VP089	image	t_succ	109.99	140.77	157.2	175.21	203.77	223.72	235.43	252.14	269.73	281.49	290.82	304.27	312.71	321.17
VP089	image	t_succ-t_init	24.9	12.06	16.43	15.22	28.56	40.26	11.71	16.71	-6.19	11.76	2.81	20.1	8.44	8.46
VP089	image	t_succ-t_preTask	24.9	12.06	16.43	15.22	28.56	19.95	11.71	16.71	-6.19	11.76	2.81	13.45	8.44	8.46
VP090	text	t_init	1533.92	1576.12	1622.38	1641.57	1647.7	1663.81	1698.66	1705.16	1764.17	1766.9	1779.77			
VP090	text	t_succ	1557.97	1622.38		1647.7	1674.2	1698.66	1705.16	1719.24	1766.9	1777.67	1803.15			
VP090	text	t_succ-t_init	24.05	46.26		6.13	26.5	34.85	6.5	14.08	2.73	10.77	23.38			
VP090	text	t_succ-t_preTask	24.05	46.26		6.13	26.5	24.46	6.5	14.08	2.73	10.77	23.38			
VP091	algorithm	t_init	52.5	93.98	108.38	129.33	133.41	151.28	161.99	167.5	207.75	210	224.09	262.83	263.36	271.1
VP091	algorithm	t_succ	62.17	108.38	119.43	133.41	152.1	161.99	167.5	178.3	210	217.4	227.95	263.36	271.1	297.35
VP091	algorithm	t_succ-t_init	9.67	14.4	11.05	4.08	18.69	10.71	5.51	10.8	2.25	7.4	3.86	0.53	7.74	26.25
VP091	algorithm	t_succ-t_preTask	9.67	14.4	11.05	4.08	18.69	9.89	5.51	10.8	2.25	7.4	3.86	0.53	7.74	26.25
VP092	text	t_init	119.26	161.63	267.53	371.44	339.98	415.65	385.71	397.27	429.76	437.32	450.75			
VP092	text	t_succ	177.55	267.53	314.42	339.98	366.69	385.71	397.27	419.87	437.32	448.74	457.28			
VP092	text	t_succ-t_init	58.29	105.9	46.89	-31.46	26.71	-29.94	11.56	22.6	7.56	11.42	6.53			
VP092	text	t_succ-t_preTask	58.29	89.98	46.89	-31.46	26.71	-29.94	11.56	22.6	7.56	11.42	6.53			
VP093	algorithm	t_init	26.97	68.67	76.15	96.75	100.52	115.97	155.32	162.18	218.96	222.77	221.95	203.09	244.4	260.52
VP093	algorithm	t_succ	37.34	76.15		100.52	130.11	155.32	162.18	172.4	222.77	229.47	236.63	244.4	260.52	271.12
VP093	algorithm	t_succ-t_init	10.37	7.48		3.77	29.59	39.35	6.86	10.22	3.81	6.7	14.68	41.31	16.12	10.6
VP093	algorithm	t_succ-t_preTask	10.37	7.48		3.77	29.59	25.21	6.86	10.22	3.81	6.7	14.68	7.77	16.12	10.6

Table 2 shows a list of the test persons (VP001 – VP093) with the number of errors during the flight and the answers to the questionnaire including the three possible instruction forms (text, algorithm, image). The columns:

– Errors unknown actions = number of errors in actions that must be executed the first time
– Errors known actions = number of errors in actions that have already been executed
– Landing on the ground = aircraft landing on the ground without crash (y = positive landing; n = aircraft involved in an accident)
– Landing at airport = landing of the aircraft on the runway (y = positive landing on the runway; n = not landing on the runway)
– Mental requirement, Physical requirement, Time requirement, Performance, Effort and Frustration = Answers from the NASA-RTLX [2, 3] and ATI-Score [4] questionnaire (Possible points 1 - 20)

**Table 2:** bh8rfvojhtio.

Test person number	Introduction form	Errors unknown aktions	Errors known aktions	Landing on the ground	Landing at airport	Mental requirement	Physical requirement	Time requirement	Performance	Effort	Frustration	Age	Sex	Flight experience	Complex system experience
V001	text	3	1	n	n	20	2	18	20	18	16	24	f	n	n
V002	algorithm	3	0	y	y	4	0	13	9	4	7	21	m	y	n
V003	image	0	0	y	y	17	2	18	4	15	4	24	m	n	n
V004	algorithm	0	0	y	y	8	5	10	4	11	7	24	m	n	n
V005	image	3	0	y	n	12	1	10	12	12	11	23	f	n	y
V006	text	1	1	y	y	6	1	15	1	7	5	25	m	y	n
V007	algorithm	3	0	n	n	14	2	16	16	15	14	28	m	y	n
V008	image	1	2	y	n	16	4	17	13	12	16	25	f	n	n
V009	text	0	0	y	y	9	5	14	5	15	14	24	f	n	n
V010	image	0	0	y	y	12	4	16	8	12	6	26	m	y	n
V011	text	3	0	n	n	14	2	10	16	12	14	23	f	n	n
V012	algorithm	3	1	n	n	16	10	18	16	16	6	24	m	n	n
V013	image	0	0	y	y	12	5	12	7	10	4	26	f	y	n
V014	text	1	0	y	n	20	0	20	10	18	14	27	f	y	n
V015	image	0	1	y	y	16	6	8	4	8	8	23	f	n	n
V016	algorithm	1	0	n	n	13	1	7	19	5	14	24	m	n	n
V017	algorithm	1	0	y	n	13	5	15	9	7	3	25	m	y	J
V018	text	2	0	n	n	16	3	19	14	10	8	38	m	y	y
V019	algorithm	3	0	y	n	17	12	18	16	14	8	22	m	n	n
V020	image	0	0	y	y	15	1	8	6	11	2	27	f	n	y
V021	text	0	0	y	y	12	6	14	13	13	8	22	f	n	n
V022	algorithm	0	0	y	y	14	2	14	4	14	5	24	m	n	n
V023	image	1	1	y	y	17	3	17	6	16	8	26	m	n	n
V024	text	0	0	y	y	14	0	16	4	14	12	32	m	n	y
V025	image	0	0	y	y	13	4	12	3	8	2	26	m	n	n
V026	text	1	0	y	y	18	1	18	3	13	14	26	f	n	n
V027	text	2	0	n	n	18	2	17	16	16	14	27	m	n	n
V028	text	1	0	y	y	14	4	12	4	10	4	23	m	n	n
V029	text	0	0	y	y	15	2	16	6	11	10	24	m	n	n
V030	algorithm	0	0	y	y	11	4	12	13	10	7	25	m	n	n
V031	algorithm	2	0	n	n	16	2	18	10	16	10	29	m	y	n
V032	image	2	0	n	n	14	5	13	10	5	13	48	m	n	n
V033	image	0	0	y	y	12	8	14	4	14	6	23	m	n	y
V034	text	2	0	y	y	10	6	9	2	10	8	20	f	n	n
V035	algorithm	1	1	y	y	2	3	14	6	2	0	20	m	y	n
V036	algorithm	1	0	y	n	12	4	10	4	14	8	22	m	y	n
V037	text	2	0	n	n	7	1	15	17	5	1	41	m	n	y
V038	image	1	1	y	y	10	2	10	4	4	0	22	m	y	n
V039	image	0	0	y	y	15	4	16	7	16	14	54	f	n	n
V040	text	1	0	y	y	18	2	14	10	4	14	18	f	n	n
V041	text	1	0	y	y	9	5	13	8	12	12	25	m	n	n
V042	algorithm	2	0	n	n	8	3	6	10	12	5	30	m	n	n
V043	text	0	0	y	y	5	4	8	2	4	1	21	m	n	n
V044	image	0	0	y	y	6	8	10	6	7	2	24	m	y	y
V045	algorithm	2	1	y	n	12	0	18	15	12	13	26	m	n	n
V046	text	2	3	n	n	7	2	12	12	10	8	21	m	n	n
V047	algorithm	0	0	y	y	7	1	10	5	3	10	22	m	n	n
V048	text	1	0	y	y	13	6	12	8	6	4	25	m	y	y
V049	text	1	0	y	y	9	5	15	15	11	7	25	m	y	y
V050	image	1	0	y	y	12	4	10	0	14	4	26	m	y	n
V051	text	1	0	n	n	16	10	14	8	16	14	25	m	n	y
V052	algorithm	2	0	n	n	12	2	14	14	6	10	28	m	n	y
V053	text	1	0	n	n	17	3	19	7	11	13	31	m	n	n
V054	image	0	0	y	y	5	2	13	4	13	5	27	m	n	n
V055	algorithm	0	0	n	n	10	4	10	3	4	0	19	m	y	n
V056	text	0	0	y	y	14	4	6	3	5	6	26	m	n	n
V057	image	0	0	y	y	6	4	10	0	6	0	24	m	y	y
V058	algorithm	0	0	y	y	18	1	16	1	16	0	22	m	n	n
V059	image	0	0	y	y	15	1	17	1	13	3	34	m	y	y
V060	image	0	0	y	y	13	1	13	1	7	1	27	m	n	n
V061	algorithm	0	0	y	y	6	6	6	6	4	4	25	m	n	y
V062	image	1	0	y	y	8	3	14	4	10	2	23	m	y	n
V063	algorithm	0	0	y	y	12	3	12	7	16	12	22	m	n	n
V064	image	0	0	y	y	11	6	14	16	8	2	30	m	n	y
V065	algorithm	0	1	y	y	14	2	13	1	8	4	26	m	y	n
V066	image	0	0	y	y	16	2	19	18	10	18	25	f	n	y
V067	algorithm	1	1	y	n	5	2	14	14	6	10	40	m	n	n
V068	image	0	0	y	y	5	1	10	2	2	1	35	m	n	y
V069	algorithm	2	1	y	n	16	4	17	15	14	8	24	m	n	n
V070	text	2	2	n	n	18	4	11	16	16	8	28	m	n	n
V071	algorithm	2	1	n	n	11	1	1	3	3	1	37	m	n	n
V072	image	0	0	y	y	11	3	11	9	7	3	47	m	n	y
V073	image	1	1	y	y	4	0	12	2	4	4	31	m	n	y
V074	algorithm	3	0	n	n	18	2	19	14	11	12	35	m	n	n
V075	text	1	0	n	n	15	3	15	9	15	11	43	m	n	y
V077	algorithm	1	0	y	y	8	4	10	12	6	6	31	m	n	n
V078	image	3	3	n	n	0	3	3	2	1	1	53	m	n	y
V079	image	0	0	y	y	12	6	14	6	8	14	21	m	y	n
V080	image	2	1	n	n	6	2	5	12	3	3	42	m	n	y
V081	algorithm	2	1	y	y	13	7	15	18	10	14	31	m	n	y
V082	text	2	2	n	n	14	2	16	0	14	0	31	f	n	n
V083	text	0	0	y	y	17	15	15	7	13	9	26	m	n	y
V084	algorithm	3	1	n	n	12	2	8	4	8	9	59	m	n	y
V085	algorithm	2	0	n	n	9	5	16	11	12	3	24	f	n	n
V086	text	1	0	y	n	11	3	17	9	5	13	39	m	y	y
V087	image	0	0	y	y	14	0	0	8	2	3	61	m	n	y
V088	image	2	0	y	n	15	6	14	7	12	10	58	f	n	n
V089	image	0	0	y	y	5	3	13	2	2	2	29	m	n	n
V090	text	2	0	n	n	14	4	15	13	14	1	15	m	n	n
V091	algorithm	0	0	y	y	14	4	14	2	14	0	26	f	y	n
VP92	algorithm	4	1	n	n	18	2	15	9	1	1	43	f	n	n
VP93	text	2	0	y	y	9	2	7	8	19	2	28	m	n	n

## 2 Experimental design, materials, and methods

### 2.1 Data

The data show the results of the flight test. These consist of the exported data of the flight research simulator (see Figs. 1 and 2) and the answers of the questionnaire about workflow of the test persons with the errors made (see Tables 1 and 2) [1].

Table 1 shows the respondents' number (VP001 - VP093), the instructions used (image, text or algorithm) and the time in seconds required to complete the 14 steps required to land the aircraft. Metric:

- t_init = time for possible start of the action
- t_succ = time for conversion of the action
- t_succ-t_init = required time for the action step
- t_succ-t_preTask = time for conversion if time too long from previous action step

Table 2 shows the respondent number (VP001 - VP093), the instructions used (image, text or algorithm), number of errors and answers from the NASA-RTLX questionnaire. Description of the columns:

- Errors unknown actions = number of errors in actions that must be executed the first time
- Errors known actions = number of errors in actions that have already been executed
- Landing on the groud = aircraft landing on the ground without crash (y = positive landing; n = aircraft involved in an accident)
- Landing at airport = landing of the aircraft on the runway (y = positive landing on the runway; n = not landing on the runway)
- Mental requirement, Physical requirement, Time requirement, Performance, Effort and Frustration = Answers from the NASA-RTLX [2, 3] and ATI-Score [4] questionnaire (Possible points 1 - 20)

Insert table 1 landscape: NASA-RTLX, Error.xlsx, Inscription: Answers form the questionnaire and number of errors.

## 3 Experimental design, materials and methods

### 3.1 Research flight simulator

The research flight simulator represents a realistic complex system and is therefore suitable for testing different representations of instructions. It has a three-channel viewing system with a viewing angle of over 180° and a Full HD resolution for each channel. The cockpit is equipped with an active Wittenstein side stick, which includes an electronic control charging system for force feedback as well as variable hard and soft stops. The SIMULINK flight system dynamics model running in the background enables data recording during test flights in the simulator.

In the scenario used, the aircraft takes off from a fixed position in the sky above Munich, a few nautical miles away from Munich Airport. The aim of the scenario is to land the aircraft safely on the runway of the airport. To successfully complete this task, a total of 14 action steps must be performed in the simulator cockpit in a specific, time-sensitive sequence. To do this, certain display values must be read and adjusted from different displays, correct inputs made to controllers, levers and buttons and certain time restrictions must be adhered to. In addition, some manual steps may only be carried out when a certain precondition has been reached. The flight scenario used is basically similar to an approach to an airport in combination with a landing by autopilot, as it is also carried out in real flight practice.

If you perform the 14 steps in the above order, the scenario ends with a "perfect" landing on the runway. With certain temporal deviations or without. This operationalisation makes it possible to check the effectiveness of the instructions for action, since only a limited number of possible outcomes are possible.

### 3.2 Introduction forms

*The same 14 steps are presented to the users in different forms. Each participant flies only once. There are n = 93 flights with 31 times image, 31 times algorithm and 31 times text.* ***Error! Reference source not found.*** *shows the first two steps in the algorithm introduction form.* ***Error! Reference source not found.*** *shows the first six steps of the text introduction and* ***Error! Reference source not found.*** *shows the eighth step of the image introduction form. The full version of the three used introductions see the digital appendix.*

## Ethics statement

No human experiments were carried out. Before the test, the test subjects signed a written declaration of consent that the data may be used anonymously for research purposes. The declaration of consent is written in German because the test subjects understand German.

## Declaration of Competing Interest

The authors Hammann, Feldhütter and Krause are full time PhD-Students at the Technical University of Munich. There is no further financial support of any companies. The equipment (Research Simulator) and software are owned by the Institute.
